# Overview of Research into mTOR Inhibitors

**DOI:** 10.3390/molecules27165295

**Published:** 2022-08-19

**Authors:** Beibei Mao, Qi Zhang, Li Ma, Dong-Sheng Zhao, Pan Zhao, Peizheng Yan

**Affiliations:** 1 College of Pharmacy, Shandong University of Traditional Chinese Medicine, Jinan 250355, China; 2 Shandong Provincial Key Laboratory of Molecular Engineering, State Key Laboratory of Biobased Material and Green Papermaking, School of Chemistry and Chemical Engineering, Qilu University of Technology (Shandong Academy of Sciences), Jinan 250353, China

**Keywords:** mTOR inhibitors, mTOR, human diseases, dual inhibitors

## Abstract

The mammalian target of rapamycin (mTOR) is a serine/threonine kinase that belongs to the phosphoinositide 3-kinase (PI3K)-related kinase (PIKK) family. The kinase exists in the forms of two complexes, mTORC1 and mTORC2, and it participates in cell growth, proliferation, metabolism, and survival. The kinase activity is closely related to the occurrence and development of multiple human diseases. Inhibitors of mTOR block critical pathways to produce antiviral, anti-inflammatory, antiproliferative and other effects, and they have been applied to research in cancer, inflammation, central nervous system diseases and viral infections. Existing mTOR inhibitors are commonly divided into mTOR allosteric inhibitors, ATP-competitive inhibitors and dual binding site inhibitors, according to their sites of action. In addition, there exist several dual-target mTOR inhibitors that target PI3K, histone deacetylases (HDAC) or ataxia telangiectasia mutated and Rad-3 related (ATR) kinases. This review focuses on the structure of mTOR protein and related signaling pathways as well as the structure and characteristics of various mTOR inhibitors. Non-rapalog allosteric inhibitors will open new directions for the development of new therapeutics specifically targeting mTORC1. The applications of ATP-competitive inhibitors in central nervous system diseases, viral infections and inflammation have laid the foundation for expanding the indications of mTOR inhibitors. Both dual-binding site inhibitors and dual-target inhibitors are beneficial in overcoming mTOR inhibitor resistance.

## 1. Introduction

The mammalian target of rapamycin, or the mechanistic target of rapamycin (mTOR), is a serine/threonine kinase that belongs to the phosphoinositide 3-kinase (PI3K)-related kinase (PIKK) family [1]. It consists of 2549 amino acids and has a molecular weight of approximately 289 kD. It is a highly conserved protein [2]. As an important part of the PI3K/protein kinase B (AKT)/mTOR signaling pathway, mTOR exists in the form of mTORC1 and mTORC2 complexes in cells, affecting protein and lipid synthesis, autophagy and the ubiquitin proteasome system. It regulates cell growth and survival and affects the formation of the actin cytoskeleton [1,3,4]. Studies have found that various diseases, such as cancer, obesity, type II diabetes, muscle diseases, and neurodegenerative diseases, are related to the abnormal expression or dysfunction of mTOR. Accordingly, mTOR may be an effective target for the treatment of multiple diseases. This article reviews a wide variety of mTOR inhibitors, hoping to provide important information for researchers.

## 2. The Structure and Function of mTOR

### 2.1. Structure of the mTOR Protein

**Figure 1:**
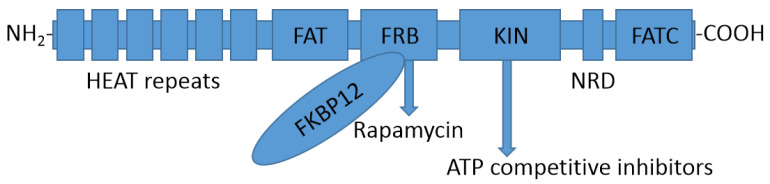
Primary structure and domain distribution of mTOR.

The mTOR protein contains multiple protein domains (Figure 1). Near the N-terminus are more than 20 repeats of Huntingtin, elongation factor 3, protein phosphatase 2A and TOR1 (HEAT) units, which form a hydrophobic surface that mediates protein–protein interactions and facilitates the localization of mTOR to the cell membrane. The next domain, a focal adhesion targeting (FAT) domain, consists of about 568 amino acids. The FAT domain forms specific interactions with a FAT domain at the C-terminus (FATC) to expose the kinase domain (KIN) [5]. The FK506-binding protein of 12 kD (FKBP12)-rapamycin binding (FRB) domain mediates interactions with the inhibitor rapamycin. After rapamycin binds to FKBP12, the FKBP12-rapamycin complex binds to the FRB domain, causing the KIN domain to undergo allosteric conformational changes, inhibiting the kinase activity. The KIN domain binds to both adenosine triphosphate (ATP) and ATP-competitive inhibitors. A serine- and threonine-rich negative regulatory domain (NRD) is located between KIN and FATC [5,6,7].

The mechanism of inhibition by rapamycin was elucidated in part through structural studies. In 1996, Kallen et al. determined the crystal structure of the binary complex of FKBP12 and 28-O-methylrapamycin (PDB ID: 4DHO) to a resolution of 2.1 Å by X-ray diffraction [8]. In the same year, Choi et al. published the crystal structure of the FKBP12-rapamycin-FRB ternary complex (PDB ID: 1FAP) with a resolution of 2.7 Å [9]. These two structures confirmed that rapamycin binds to the FRB domain after forming a complex with FKBP12 protein.

The analyses of crystal structures of mTOR has provided information that has supported the discovery and optimization of highly active and highly selective mTOR inhibitors. A major breakthrough in the structural analysis of mTOR was achieved in 2013. Yang et al. determined the co-crystal structure of a complex composed of an mTOR N-terminal truncation mutant (mTOR^ΔN^), mammalian lethal with sec-13 protein 8 (mLST8) and a non-hydrolyzable ATP analog (ATPγS·Mg^2+^). In addition, they also solved the co-crystal structures of mTOR^ΔN^-mLST8 with three inhibitors that compete at the ATP binding site, Torin2, PP242 and PI-103, to resolutions of 3.2 to 3.6 Å. In each case, the structural models of mTOR^ΔN^-mLST8 encompass approximately 1500 amino acids, and contain the complete FAT, FRB, KIN, FATC domains and the mLST8 structure. The structure of the protein complex is compact, with the FAT domain exhibiting multiple α-helices that form a C-shaped solenoid that sandwiches and almost completely encloses the KIN domain. The mLST8 and FRB domains extend from the KIN domain in opposite directions toward the catalytic cleft. FATC was found to be integral to the overall structure of the kinase domain [10].

### 2.2. Signaling Pathway and Function of mTOR

#### 2.2.1. The Composition of mTORC1 and Its Signaling Pathway

The complex mTORC1 consists of mTOR and related proteins Raptor, mLST8, proline-rich AKT substrate 40 kDa (PRAS40), and DEP domain containing mTOR-interacting protein (Deptor) [11,12,13]. As a backbone protein, Raptor regulates the assembly, localization and binding of mTORC1 to substrates. As endogenous inhibitors of mTORC1, PRAS40 and Deptor negatively regulate the kinase activity of the complex [1]. 

**Figure 2:**
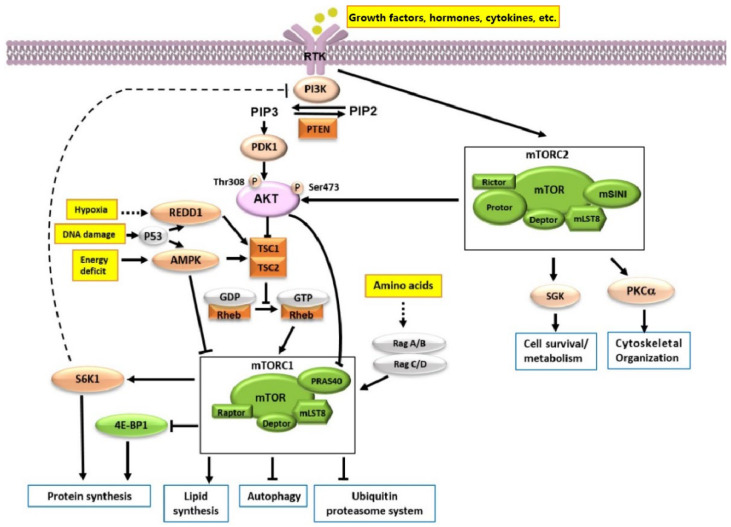
mTOR signaling pathways. mTORC1 responds to extracellular growth factors, stress, oxygen, and amino acids. Growth factors, hormones, cytokines, etc., activate PI3K, activated PI3K phosphorylates PIP2 to form PIP3, then AKT is phosphorylated and activated. AKT activates mTORC1 by inhibiting the interaction between TSC-1 and TSC-2. Low energy, hypoxia, and DNA damage can inhibit the activity of mTORC1 by activating REDD1 or AMPK, and inhibiting Rheb. The activation of mTORC1 by amino acids mainly occurs because Rag dimers are activated to form RagA/B and RagC/D. The signals downstream of mTORC1 activation mainly involve S6K1 and 4E-BP1, which affect synthesis of proteins and lipids. mTORC1 also affects autophagy and ubiquitin proteasome system. Signals downstream of mTORC2 regulate AKT, SGK1 and PKC, which regulate cell metabolism, survival and cytoskeletal organization.

The complex responds to various stimuli, such as extracellular growth factors, stress, oxygen, and amino acids (Figure 2). Growth factors, hormones, cytokines, etc., activate mTORC1 directly or indirectly through PI3K and AKT [1,14]. Activated PI3K phosphorylates phosphatidylinositol (4,5)-biphosphate (PIP_2_) to form phosphatidylinositol (3,4,5)-triphosphate (PIP_3_), which binds to the pleckstrin homology (PH) domain of AKT [15]. This membrane-associated AKT is phosphorylated and activated, and it both directly activates mTORC1 and indirectly activates mTORC1 by inhibiting the interaction between tuberous sclerosis 1 (TSC-1) and tuberous sclerosis 2 (TSC-2). Dimerized TSC-1/TSC-2 inhibits the GTPase Ras-homolog enriched in brain (Rheb), which is required for mTOR activation; therefore, TSC-1/TSC-2 normally inhibits mTOR. However, activated AKT phosphorylates Ser939 and Thr1462 of TSC-2, preventing the formation of the TSC-1/TSC-2 complex and relieving the inhibition of Rheb [16,17,18].

The mTORC1 complex responds to stressors. Low energy, hypoxia, and DNA damage, for example, can inhibit the activity of mTORC1. Adenosine monophosphate (AMP)-activated protein kinase (AMPK) directly or indirectly inhibits mTORC1 during energy starvation. AMPK is sensitive to changes in intracellular AMP levels and thus can be considered an energy sensor. When a cell is energy-deficient, AMPK is activated by high AMP levels and can inhibit mTORC1 by phosphorylating the complex, or it can increase the activity of TSC-2 by promoting the formation of the TSC-1/TSC-2 complex, thereby indirectly inhibiting the activity of mTORC1 [19,20,21]. When cells are in a hypoxic environment, the activity of mTORC1 is inhibited mainly through a pathway involved in regulating development and DNA damage response 1 (REDD1) and TSC1/TSC2 [22,23]. DNA damage can trigger p53-dependent transcription, increase TSC2 activity by activating REDD1 or AMPK, and inhibit Rheb, thereby inhibiting mTORC1 [24,25].

The activation of mTORC1 by amino acids mainly occurs due to stimulation by leucine, arginine and glutamine, which affect the localization of lysosome vacuolar H^+^-ATPase (v-ATPase) and regulate the binding of the small GTPase Rag to guanine nucleotides. This regulation affects the binding of Rag to mTORC1, which promotes mTORC1 activation by Rheb [26,27]. When stimulated by amino acids, Rag dimers localized to the lysosomal surface via lysosomal v-ATPase are activated by guanylate exchange factor (GEF) to form RagA/B and RagC/D. These conformations bind to GTP and guanosine diphosphate (GDP), respectively. At this point, active, GTP-bound Rag binds to Raptor in the mTORC1 complex and recruits mTORC1 to the lysosome surface, where mTORC1 is further activated when it encounters GTP-bound Rheb [28].

The signals downstream of mTORC1 activation mainly involve the regulation of ribosomal 40S small subunit S6 kinase 1 (S6K1) and eukaryotic translation initiation factor 4E (eIF4E)-binding protein 1 (4E-BP1), and it has important effects on autophagy and the ubiquitin-proteasome system. The mTORC1 complex regulates protein translation through direct kinase substrates S6K1 and 4E-BP1 [29]. The phosphorylation of ribosomal S6 protein by S6K1 regulates the initiation of 5′ terminal oligopyrimidine tract mRNA translation protein (5′ TOP). Thus, activation of S6K1 by mTOR facilitates the binding of the ribosomal 40S small subunit to the translation complex and increases the efficiency of the translation of 5′ TOP mRNAs [30,31].

The mTOR substrate 4E-BP1 is a negative regulator of translation. When activated, it binds to the mRNA5′ cap-binding protein eIF-4E and inhibits its activity, thereby inhibiting the initiation of translation. However, activated mTOR can phosphorylate and inactivate 4E-BP1. After inactivation, 4E-BP1 dissociates from eIF-4E, and eIF-4E combines with eIF-4G, eIF-4B and eIF-4A to form an eIF-4F complex, which binds to the cap structure at the end of mRNA5′ and promotes translation initiation [32,33]. In addition, eIF-4E can also regulate cyclin D, Myc, Ras, and other proteins, thereby affecting cell proliferation and cell cycle [34].

Activation of mTORC1 inhibits autophagy at multiple levels. The autophagy initiation complex is inhibited by mTORC1 through phosphorylation of the autophagy-related protein autophagy-related gene 13 (ATG13) and the unc-51-like kinase 1/2 (ULK1/2) complex. Similarly, the mTORC1 complex also inhibits autophagy by affecting the stability of ULK1 by inhibiting the phosphorylation of autophagy/beclin 1 regulator 1 (AMBRA1) [35]. It affects vesicle transport and inhibits autophagy by phosphorylating ATG14L in the vacuolar protein sorting 34 (VPS34) complex. Autophagy is also inhibited at the transcriptional level by the regulation of the transcriptional regulator transcription factor EB (TFEB) and the blocking of the expression of lysosomes and autophagy genes [36].

Proteolysis induced by the inhibition of mTORC1 occurs not only through autophagy, but also through the activation of the ubiquitin-proteasome system. This proteolysis process completed by activating the ubiquitin-proteasome system is independent from the transcription, translation, synthesis, autophagy and other processes in which mTOR participates. In this process, mTORC1 increases ubiquitination by stimulating the production of Lys48-linked ubiquitinated proteins. This process is mainly involved in the degradation of long-lived proteins, including some growth-related proteins, such as cytoplasmic 3-hydroxy 3-methyl glutaryl CoA (HMGCoA) synthase (HGMCS1), suppressor of Ty homologue-6 (SUPT6H), α-taxilin and Myst2 [37].

#### 2.2.2. The Composition of mTORC2 and Its Signaling Pathway

The complex mTORC2 includes mTOR, the rapamycin-insensitive companion of mTOR (Rictor), mLST8, Deptor, mammalian stress-activated map kinase-interacting protein 1 (mSIN1) and protein observed with Rictor (Protor) [3]. Rictor acts as a backbone protein that regulates mTORC2 assembly and substrate binding. mSIN1 also acts as a backbone protein that regulates the assembly of mTORC2 and also regulates interactions with the substrate serum- and glucocorticoid-induced protein kinase 1 (SGK1) [38]. Protor favors increased mTORC2-mediated activation of SGK1, whereas Deptor negatively regulates mTORC2 and is the only known mTORC2-specific endogenous inhibitor [39]. The presence of mLST8 is required to maintain mTORC2 activity [1].

Additional signals downstream of mTORC2 regulate some members of the AGC family of kinases, including AKT, SGK1 and protein kinase Cα (PKCα). AKT is directly phosphorylated by mTORC2 at Ser473 in the regulation of cell metabolism, survival, apoptosis, growth and proliferation [40]. The complex also controls ion transport and growth by activating SGK1 [41], and its activation of PKCα affects the actin cytoskeleton and regulates cell shape [3,40].

## 3. Inhibitors of mTOR

Existing mTOR inhibitors are divided into three categories according to their sites of action: (1) allosteric inhibitors of mTOR that bind to the FRB domain; (2) ATP-competitive mTOR inhibitors that bind to the KIN domain; and (3) dual binding site inhibitors. In addition to these mTOR inhibitors, dual-target mTOR inhibitors are also summarized here.

### 3.1. Allosteric Inhibitors of mTOR

#### 3.1.1. Rapamycin and Its Derivatives

**Figure 3:**
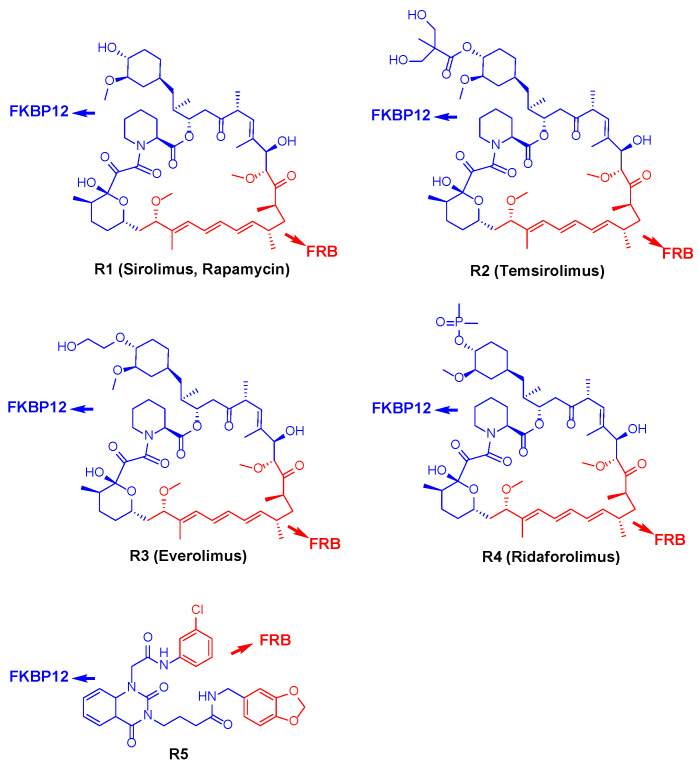
Structures of allosteric inhibitors of mTOR and representations showing the binding mode of them with FKBP12 (blue) and FRB (red). **R1**–**R4**: rapamycin and its derivatives; **R5**: non-rapalog allosteric inhibitor.

Rapamycin (**R1**, Figure 3), also known as sirolimus, was the first known mTOR inhibitor and was originally isolated from *Streptomyces hygroscopicus*. It was approved by the U.S. Food and Drug Administration (FDA) in 1999 as an immunosuppressant for the prevention of organ transplant rejection. However, its poor solubility and low bioavailability have limited its further development. Rapamycin derivatives, such as temsirolimus (**R2**), everolimus (**R3**), and ridaforolimus (**R4**), retain the backbone structure of rapamycin but contain modifications at the C-42 position that improve solubility and pharmacokinetic properties. This type of inhibitor binds to FKBP12 and then forms a ternary complex with the FRB domain of mTOR, allosterically altering the active site of mTOR and inhibiting the kinase activity [42].

In 2007, temsirolimus was approved by the FDA for the treatment of advanced renal cell carcinoma. Everolimus, initially used as an immunosuppressant, was also approved for the treatment of oncological diseases, including advanced renal cell carcinoma, subependymal giant cell astrocytoma, and tuberous sclerosis [43], and later it was also used in combination with exemestane in the treatment of breast cancer [44]. Oral administration of ridaforolimus resulted in significant improvement in patients with metastatic soft tissue or osteosarcoma as compared with placebo [45].

#### 3.1.2. Non-Rapalog Allosteric Inhibitor

Using an in silico and in-cell hybrid strategy that simultaneously targets both FKBP12 and the FRB domain of mTOR, Shams et al. developed a non-rapalog allosteric inhibitor **R5**. **R5** significantly inhibits the phosphorylation of mTORC1 substrates S6K1 and 4E-BP1 with an IC_50_ of 10 nM for ^T389^p-S6K1, resulting in cancer cytotoxicity, and it inhibits the proliferation of HeLa cells with a IC_50_ of 3.5 nM. In addition, **R5** inhibits tumor growth in vivo without promotion of metastasis [46].

Allosteric inhibitors **R1**–**R5** formed ternary complexes with FKBP12 and FRB domain of mTOR, resulting in the allosteric inhibition of mTORC1. Rapamycin and its derivatives, called rapalogs, only affect the phosphorylation of S6K1 and do not inhibit the phosphorylation of substrate 4E-BP1. The non-rapalog allosteric inhibitor **R5** inhibited the phosphorylation of not only S6K1 but also 4E-BP1. This novel allosteric inhibitor provides a platform for the development of new therapeutic agents for cancer treatment.

### 3.2. ATP Competitive Inhibitors

Although traditional rapalogs have been used in the treatment of various tumors, they have the limitation of incomplete inhibition of mTORC1 and a lack of inhibition of mTORC2. ATP-competitive mTOR inhibitors directly act on the ATP-binding pocket of the catalytic domain of mTOR kinase and can inhibit both mTORC1 and mTORC2, leading to potential therapeutic advantages. Such inhibitors thus have become the focus of mTOR inhibitor research. ATP-competitive mTOR inhibitors can be divided into four categories, according to the structural backbones of the compounds.

#### 3.2.1. Morpholine-Substituted Heterocyclic Skeleton Inhibitors

**Figure 4:**
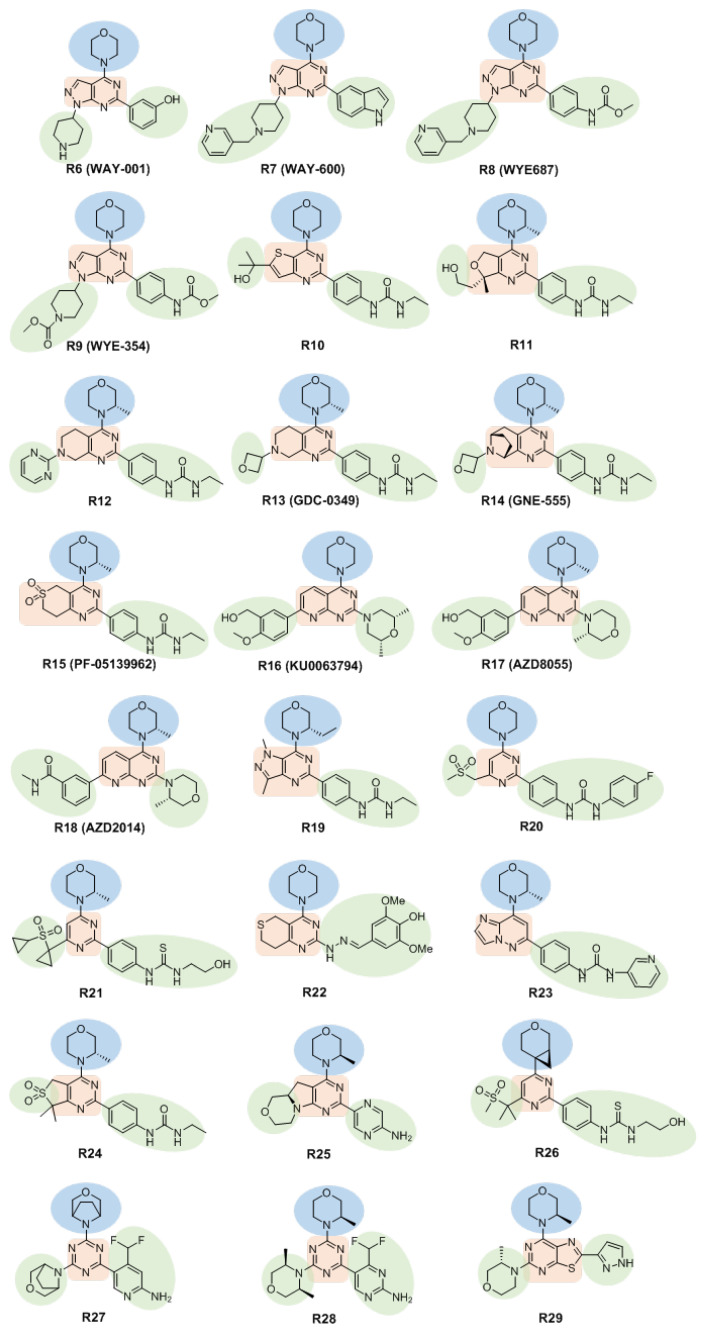
Structures of morpholine-substituted heterocyclic skeleton inhibitors. They are described as scaffold involving three regions: the hinge region (blue), the central region (red) and variable regions (green).

Yu et al. discovered the mTOR inhibitor **WAY-001** (**R6**, IC_50_ = 220 nM, Figure 4) by high-throughput screening. The mTOR-selective inhibitors **WAY-600** (**R7**, IC_50_ = 9.0 nM), **WYE-687** (**R8**, IC_50_ = 4.6 nM) and **WYE-354** (**R9**, IC_50_ = 4.3 nM) were obtained through further optimization of 4-morpholinepyrazolo [3,4-*d*]pyrimidine compounds, which have been shown to inhibit the phosphorylation of S6K1 at Thr389 and AKT at Ser473 in cells, but have been found to not inhibit the phosphorylation of AKT at Thr308. The above results indicated that compounds **R7**, **R8**, and **R9** exhibited mTOR inhibitory activity at both the enzymatic and cellular levels [47].

Genentech Inc. (South San Francisco, CA, USA) reported an mTOR inhibitor, **R10** (IC_50_ = 3 nM). The inhibitory activity of this compound against mTOR was 20-fold higher than for PI3Kα. Optimization of the skeleton of compound **R10** resulted in compound **R11**, which inhibited mTOR and PI3Kα kinases with IC_50_ values of 0.7 nM and 80.5 nM, respectively [48]. Part of the substituent group was further optimized to obtain compounds **R12**–**R14** [48,49,50,51], all of which showed good mTOR kinase selectivity. Because compound **R12** showed a time-dependent effect on cytochrome P450 3A4 (CYP3A4), the compound **GDC-0349** (**R13**) was designed and synthesized and it showed better plasma clearance and lacked the time-dependent inhibition of CYPs. In addition, compound **R13**, administered as a single drug or in combination, showed efficacy in mouse xenograft cancer models [50]. Further optimization of compound **R13** gave compound **GNE-555** (**R14**), which further weakened the time-dependent inhibition of CYP3A4, improved the water solubility and oral absorption rate, decreased the drug clearance rate, and improved the potency and selectivity of the inhibition of mTOR; it is a highly selective and metabolically stable mTOR inhibitor [51]. Similarly, Pfizer Inc. (New York, NY, USA) designed and synthesized the compound **PF-05139962** (**R15**) by modifying part of **R10**, and **R15** was found to be more than 500-fold more selective for mTOR kinase than PI3Kα, and it showed efficacy at the cellular level with good in vitro pharmacokinetics. However, the efficacy and PK performance in vivo were not sufficient to proceed with further studies [52].

Among a series of 4-morpholinopyrido [2,3-*d*]pyrimidine mTOR inhibitors reported by AstraZeneca Inc. (London, UK) in 2009, **Ku-0063794** (**R16**) was selective for mTOR relative to other kinases of the PIKK family, and it showed no detectable inhibitory activity against 200 non-PI3K-related kinases at a concentration of 10 µM. **R16** inhibits the phosphorylation of mTOR substrates rS6 (Ser235/236) and AKT (Ser473) in U87MG cells with IC_50_ values of 0.10 µM and 0.15 µM, respectively [53]. However, **R16** has some disadvantages, such as insufficient inhibitory potency on cell proliferation, low solubility, and strong inhibition of the human K_V_11.1 potassium channel (hERG). To improve these deficiencies, further optimization resulted in compounds **AZD8055** (**R17**) and **AZD2014** (**R18**) [54]. **R17** is a potent mTOR inhibitor (IC_50_ = 0.13 nM). It is also selective; the activity of **R17** against mTOR is more than one million-fold higher than against PI3K kinase. **R17** inhibits the phosphorylation of mTOR (Ser2448/2481), rS6 (Ser235/236), S6K1, 4E-BP1 (Thr37/46) and AKT (Ser473) in vitro and in vivo. A dose-dependent tumor growth inhibitory effect was shown in a mouse xenograft model [54]. In addition, **R17** inhibits the phosphorylation of the autophagy-initiating protein kinase ULK1. It can be used in combination with the mitogen-activated protein kinase kinase (MEK) inhibitor **selumetinib** to induce apoptosis and inhibit tumor growth [55,56,57]. However, it exhibits high liver toxicity in human subjects and may present other risks. **R17** entered clinical Phase I and Phase II trials in 2009, but clinical trials were withdrawn in 2011 for unreported reasons. **R18** is an optimized version of **R17**, and it is also a more potent and selective inhibitor of mTOR kinase with an IC_50_ of 2.8 nM, which is 1000-fold lower than that for PI3K. **R18** has the characteristics of high cell proliferation inhibitory efficacy, low hepatotoxicity, good solubility, an acceptable IC_50_ for hERG, and consistent efficacy in rodents [54]. This compound has entered Phase II clinical trials.

The introduction of an *N*-7-methyl group into the imidazopyrimidine structure has proven to improve the selectivity of the compound for mTOR. For example, Lee et al. designed and synthesized a series of mTOR-selective inhibitors by introducing *N*-7-methyl groups into pyrrolo [3,2-*d*]pyrimidine and pyrazolo [4,3-*d*]pyrimidine rings. Among them, the pyrazolo [4,3-*d*]pyrimidine compound **R19** exhibits a K_i_ value of 2 nM for mTOR, which is more than 2900-fold better than its K_i_ for PI3K, and **R19** has been shown to effectively inhibit cell proliferation [58].

The sulfonylmorpholine-substituted pyrimidine aryl urea compound **R20** is a selective mTOR inhibitor developed by AstraZeneca Inc. (London, UK) It exhibits good inhibitory activity at both the kinase level and the cellular level. **R20** showed good selectivity, with IC_50_ values of 28 nM and 565 nM for mTOR and PI3Kα, respectively [59]. Developed on the basis of this structure, compound **R21** was found to have improved efficiency, water solubility and stability in human hepatocytes, and it can efficiently and selectively inhibit the activities of mTORC1 and mTORC2, with good physicochemical properties and pharmacokinetics. It has the potential to be developed as a clinical drug [60].

Zhu et al. designed and synthesized a series of 7,8-dihydro-5*H*-thiopyrano [4,3-*d*]pyrimidine derivatives, all of which exhibited mTOR inhibitory activity at a concentration of 10 µM. The IC_50_ values of compound **R22** against mTOR kinase, H460 cells and PC-3 cells were 0.80 µM, 7.43 µM and 11.90 µM, respectively [61].

The research group of Zhang designed and synthesized a series of imidazo [1,2-*b*]pyridazine derivatives through skeleton transformation, and obtained compound **R23**, which demonstrated mTOR inhibitory activity with an IC_50_ of 67 nM. Compound **R23** showed a clear inhibitory effect on the proliferation of A549 cells, with an IC_50_ of 50 nM. Compound **R23** inhibits the phosphorylation of downstream proteins AKT and S6 in cells, arrests the cell cycle in G1 phase, and exhibits in vivo antitumor activity in a tumor xenograft A549 model in nude mice [62].

Cansfield et al. designed and synthesized compound **R24**, with a morpholine-substituted pyrimidosulfone ring. As a highly selective mTOR inhibitor with favorable in vitro and in vivo pharmacokinetic properties, it exhibits potent in vivo anti-inflammatory activity in a collagen-induced arthritis model. This is the first reported mTOR inhibitor with anti-inflammatory activity [63].

Starting with a compound with pan PIKK activity, Borsari et al. used conformational restriction to enhance the potency and selectivity of mTOR inhibition, and obtained an ATP-competitive inhibitor, compound **R25** (IC_50_ = 8 nM), with a tricyclic pyrimidine-pyrrole-oxazine backbone. Compound **R25** has 450-fold stronger inhibition against mTOR relative to PI3K, and it has high oral bioavailability, metabolic stability, and minimal brain permeability and therefore has potential applications in the treatment of systemic tumors [64].

Hobbs et al. found that 3-oxabicyclo [4.1.0]heptane can be used as a potent non-nitrogen-containing morpholine isostere that replaces the morpholine structure. Cyclopropylpyridine analog **R26** retains affinity for mTOR (pIC_50_ = 8.6 nM) and cytostatic potency. Compound **R26** exhibits higher selectivity and similar solubility compared to class I PI3K analogs [65]. 

The laboratory of Wymann obtained a novel, potent and selective mTOR inhibitor, **R27**, by introducing difluoromethyl substituents and 3,5-ethylene bridged morpholines into pan-PI3K/mTOR inhibitors. **R27** exhibits strong selective inhibition of mTOR kinase (IC_50_ = 10.8 nM), exhibits antitumor effects in vitro and in vivo, and exhibits advantages in central nervous system indications due to its excellent brain/plasma partitioning [66]. The morpholine base of compound **R27** was further modified to obtain compound **R28**, and its metabolic stability in the human body and inhibitor potency (IC_50_ = 3.6 nM) were improved. Compound **R28** shows excellent brain penetration ability, is well tolerated in mice, and overcomes the metabolic defects exhibited by **R27**, and it became the first mTOR kinase inhibitor shown to be effective in a mouse model of tuberous sclerosis complex (TSC) [67].

Bonazzi et al. optimized the physicochemical space of purine derivative mTOR inhibitors based on the key parameters of central nervous system drugs, and obtained a thiazolidine-based mTOR kinase inhibitor, compound **R29** (pS6 IC_50_ = 5.6 nM) for central nervous system (CNS) indications. Inhibitor **R29** exhibited good kinase selectivity and good brain exposure and significantly improved survival in TSC model mice [68].

Morpholine-substituted heterocyclic skeleton inhibitors can be structurally described as scaffold involving three regions (Figure 4). In the hinge region, nitrogen or oxygen atoms will help improve the interaction and strength. It is not hard to find out that all compounds have a morpholinyl group in the hinge region with the exception of compound **R26** (replaced morpholine structure with 3-oxabicyclo [4.1.0]heptane). Indeed, the introduction of the morpholine motif into the structure of potential inhibitors has been proved to be beneficial for mTOR inhibition. The central region connects the hinge region and variable regions together. The central region is located in a relatively large space and can therefore be represented by structurally variable motifs. This class of mTOR inhibitors also consists of one or two additional variable regions for improving selectivity and modulating the physicochemical properties.

#### 3.2.2. Inhibitors Based on Quinoline Structures

**Figure 5:**
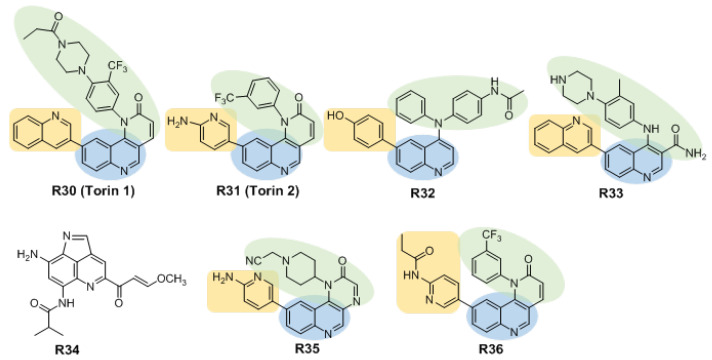
Structures of inhibitors based on quinoline. They are described as scaffold involving three regions: the hinge region (blue), the hydrophobic region (buff) and variable region (green). **R34** is not described.

Liu et al., starting from a quinoline structure with mTOR inhibitory activity, obtained the compound **Torin1** (**R30**, Figure 5) through structural modification. A six-membered cyclic lactam is an essential part of its activity; changing this lactam to a cyclic urea, carbamate or pyrimidinedione structure leads to a reduction in inhibitory activity of more than 500 fold. In addition, the introduction of a methyl group at the α or β position of the lactam ring significantly reduces the potency of the compound in biochemical and cellular experiments. **R30** inhibits the phosphorylation of mTORC1 and mTORC2 in cells at concentrations of 2 nM and 10 nM, respectively [69]. The compound is approximately 1000-fold more active against mTOR than against PI3K, and the anti-mTOR activity is at least 100 fold higher than its activity against 450 other protein kinases. However, poor solubility, short half-life, and low oral bioavailability limited the further development of **R30**. As a continuation of **R30**, the compound **Torin2** (**R31**) [70], obtained by removing a propionylpiperazine group and replacing the aminopyridine moiety with quinoline, has good mTOR inhibitory activity and selectivity, and shows good bioavailability (54%) and metabolic stability [71].

Compounds known as 4-(*N*-phenyl-*N′*-substituted benzenesulfonyl)-6-(4-hydroxyphenyl)quinolines were designed and synthesized by Venkateswarlu et al. and exhibited strong mTOR inhibitory activity at a concentration of 0.5 µM. Among these compounds, compound **R32** exhibited an IC_50_ of 613 nM for mTOR and caused cell cycle arrest in MiaPaCa-2 cells [72].

The quinoline derivatives were designed and synthesized by introducing intramolecular hydrogen bonds, which led to inhibitory activity against mTOR and proliferation inhibitory activity against HCT-116, PC-3 and MCF-7 cells. A representative compound, **R33**, inhibits mTOR activity with an IC_50_ of 14 nM, and its proliferation activities against HCT-116, PC-3 and MCF-7 cell lines occur with IC_50_ values of 0.46, 0.61 and 0.24 µM, respectively [73].

Miyanaga et al. obtained lymphostin analogs of pyridinequinoline alkaloids with potent mTOR inhibitory activity through biosynthesis in *Salinispora* bacteria. The enzyme inhibitory activity IC_50_ of representative compound **R34** was 0.8 nM, and the IC_50_ values of inhibition of the human prostate cancer cell line LNCap and human breast cancer cell line MDA-MB-468 were 22 nM and 58 nM, respectively [74].

Guo et al. obtained the pyrazino [2,3-*c*]quinolin-2(1*H*)-one scaffold as a hit compound by high-throughput screening. On the basis of this structure, a highly selective and effective oral mTOR inhibitor, **R35**, was obtained. It has high selectivity, and the inhibitory activity of compound **R35** on mTOR reaches 7 nM. In vitro, compound **R35** exhibited potent antitumor activity against human breast and cervical cancer cells, and induced tumor cell cycle arrest and autophagy. In the T-47D mouse xenograft model, oral administration of **R35** led to regression of the tumor without obvious toxicity [75].

Hand-foot-and-mouth disease (HFMD) is an infectious disease caused by a variety of enteroviruses, especially enterovirus 71 (EV71). There are currently no effective antiviral drugs or preventive vaccines against EV71. After the EV71 virus attaches and enters the host cell, it activates the PI3K/Akt/mTOR signaling pathway, reduces the apoptosis of host cells caused by virus infection, and enables the virus to continue to infect cells. In recent years, studies on virus inhibition by inhibiting mTOR in host cells have been widely reported. Based on the existing mTOR inhibitor **R31**, Hao et al. designed and synthesized 30 new derivatives to improve water solubility. Their anti-EV71 activity was evaluated, and it was found that the highly effective compound **R36** targeting mTOR had an IC_50_ value of 27 μM for inhibiting EV71, which was the closest to the IC_50_ value of **R31**. In addition, compound **R36**, which is 15-fold more water-soluble than **R31**, also was found to strongly inhibit mTOR (IC_50_ = 29 nM) [76].

Inhibitors based on quinoline are C-shaped compounds, involving three regions (Figure 5). Quinoline groups are structurally important in the hinge region to promote hydrogen bonding interactions. In contrast to the inhibitors of morpholine-substituted, this class of mTOR inhibitors have aromatic, heteroaromatic or fused ring groups in the hydrophobic region. The hydrophobic region is located in proximity to the hinge region, which is suitable for promoting interactions with nonpolar or hydrophobic groups. Thus, nonpolar and hydrophobic groups are beneficial in this region. The variable region is connected to the hinge region, and the design motifs of the variable regions are structurally changeable.

#### 3.2.3. Inhibitors Based on Pyrazolo [3,4-d]pyrimidin-4-amine and Its Derivatives

**Figure 6:**
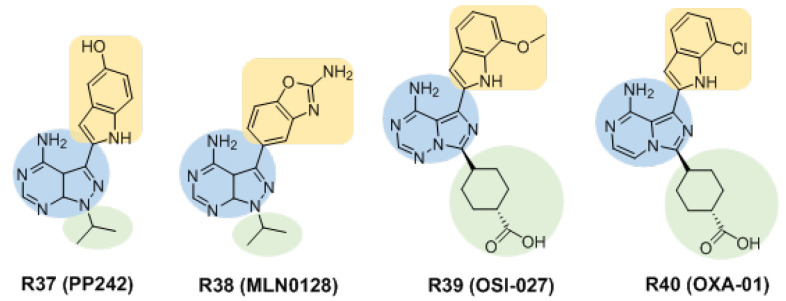
Structures of inhibitors based on pyrazolo [3,4-*d*]pyrimidin-4-amine. They are described as scaffold involving three regions: the hinge region (blue), the hydrophobic region (buff) and variable region (green).

The compound **PP242** (**R37**, Figure 6) was the first selective ATP-competitive mTOR inhibitor that was shown to inhibit the phosphorylation of AKT (Ser473), 4E-BP1, S6K1 and rS6 [77]. **MLN0128** (**INK-128**, **R38**) is a derivative of compound **R37** developed by Intellikine Inc., and it is currently in Phase II clinical trials. **R38** is a potent and highly selective mTORC1/2 (mTOR IC_50_ = 1 nM) inhibitor with drug-like properties. In vitro and in vivo experiments showed that **R38** could simultaneously inhibit the phosphorylation of AKT (Ser473), S6K1 and 4E-BP1. The results of xenograft model experiments show that oral administration of **R38** can inhibit tumor angiogenesis and tumor growth [78]. Studies have shown that compared with rapamycin, **R38** is more effective in patients with tuberous sclerosis-related tumors [79]. Another study showed that **R38** inhibited B-cell acute lymphoblastic leukemia and reduced colony formation [80].

**OSI-027** (**R39**) is an analog of pyrazolo [3,4-*d*]pyrimidin-4-amine optimized from an imidazo [1,5-*a*]pyrazine lead by Crew et al. [81]. The IC_50_ of **R39** for mTORC1 and mTORC2 is 22 nM and 65 nM, respectively, and it exhibits a higher than 100-fold selectivity for mTOR relative to PI3K*α*, PI3K*β*, PI3K*γ* and DNA-PK. In a variety of cancer models, **R39** was found to completely inhibit the phosphorylation of 4E-BP1, S6K1 and AKT, and it showed good oral and pharmacodynamic effects and highly potent antitumor effects in a xenograft model that was resistant to rapamycin [82]. **R39** has completed Phase I clinical trials for solid tumors and lymphomas. **OXA-01** (**R40**) has been developed as another pyrazolo [3,4-*d*]pyrimidin-4-amine analog, with IC_50_s of 4 nM and 190 nM for mTOR and PI3K, respectively. The highly potent inhibitory effect of **R40** on some cell lines is related to its ability to completely block the phosphorylation of 4E-BP1 (Thr37/46) [83].

Similar to the inhibitors based on quinoline, pyrazolo [3,4-*d*]pyrimidin-4-amine derivatives are also C-shaped compounds, involving three regions (Figure 6). The pyrazolo [3,4-*d*]pyrimidin-4-amine skeleton is located in the hinge region. Various fused-heterocycles are located in the hydrophobic region, facilitating interactions with nonpolar or hydrophobic groups and the variable region is a changeable design site.

#### 3.2.4. Other Structural Skeletal Inhibitors

**Figure 7:**
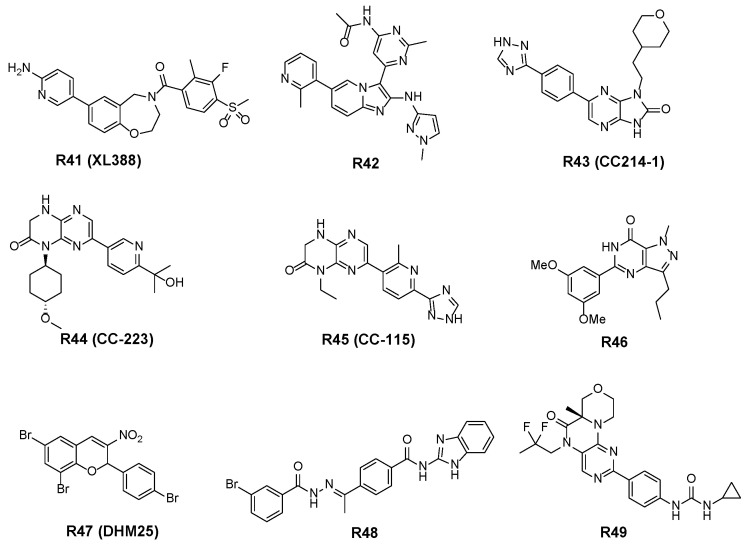
Structures of other structural skeletal inhibitors.

Takeuchi et al. discovered **R41** (Figure 7), a novel structural type of mTOR inhibitor. **R41** selectively inhibits mTOR activity with IC_50_ values of 8 nM and 166 nM for mTORC1 and mTORC2, respectively. **R41** inhibits the phosphorylation of p70S6K in MCF-7 cells with an IC_50_ value of 94 nM, and inhibits the phosphorylation of AKT (Ser473) with an IC_50_ of 350 nM. Administration of **R41** alone or in combination with paclitaxel or doxorubicin in cells and in vivo can inhibit tumor growth [84].

The imidazopyridine mTOR inhibitor **R42** was found by Peterson et al. of Amegen Inc., and it is highly selective for mTOR, with IC_50_ values of 15 and 4.3 μM for mTOR and PI3Kα kinases, respectively [85].

The selective mTOR inhibitor **R43**, designed and synthesized by Mortensen et al., exhibits IC_50_ values of 2 and 1.38 μM for mTOR and PI3Kα, respectively. It has a significant inhibitory effect on PC-3 cell proliferation, with an IC_50_ of 224 nM, and good antitumor activity in vivo was shown in a PC-3 cell xenograft model [86]. Further structure–activity relationship studies on **R43** found that 3,4-dihydropyrazino [2,3-*b*]pyrazin-2(1*H*)-one compounds **CC-223** (**R44**) [87] and **CC-115** (**R45**), showed good pharmacokinetic properties and efficient inhibition against mTORC1 (pS6) and mTORC2 (pAKTS473) in vitro and in vivo. In a xenograft model test, a dose-dependent in vivo efficacy was shown [88]. Currently, **R45** is in Phase I clinical trials, and **R44** is in Phase II clinical trials.

Reddy et al. obtained a series of 1*H*-pyrazolo [4,3-*d*]pyrimidin-7(6*H*)-ones by microwave-assisted reactions, and the group evaluated the effects of these compounds on the proliferation of HeLa, CAKI-I, PC-3, MiaPaca -2 and A549 cells. The active compound **R46**, with an IC_50_ of 203 nM for mTOR, can inhibit the phosphorylation of downstream proteins eIF4E and p70S6k, cause cell cycle arrest and induce apoptosis [89].

Fouque et al. designed and synthesized a series of benzopyran derivatives, and obtained the mTOR inhibitor **R47**, which has a strong antiproliferative effect on breast cancer tumor cells. Notably, biochemical and cellular analyses, modeling and other experimental data demonstrated that **R47** is a selective covalent inhibitor of mTOR. In vivo, **R47** can effectively inhibit the growth and metastasis of triple-negative breast cancer (TNBC) cells, leading to good development prospects [90].

Xu et al. used 3-bromo-*N’*-(4-hydroxybenzylidene)-4-methylbenzohydrazide as a lead compound and simplified the structure through pharmacophore-based virtual screening to find the novel mTOR small molecule inhibitor, **R48**, and showed that it inhibited mTOR with an IC_50_ of 304 nM. Compound **R48** can induce autophagic death and apoptosis in MDA-MB-231 and MDA-MB-468 cells and inhibit the proliferation of TNBC cells [91].

Jin et al. adopted a structure-based drug discovery strategy to rapidly design a series of tetrahydro-[1,4]oxazino [3,4-*h*]pteridin-6(5*H*)-one mTOR inhibitors. Among them, compound **R49** has the best mTOR inhibitory activity, with a pIC_50_ of 7.9, and it is selective, with a 100-fold better inhibitory activity against mTOR relative to PI3K. At the same time, this compound also exhibits excellent in vitro and in vivo efficacy and demonstrated a safe profile in absorption, digestion, metabolism and excretion studies [92].

ATP-competitive mTOR inhibitors act on the ATP-binding site of KIN domain of mTOR, and can inhibit both mTORC1 and mTORC2, offering a greater therapeutic potential than allosteric inhibitors. At present, most of the ATP-competitive mTOR inhibitors are in the preclinical research stage at the cellular and animal levels (Table 1). Several ATP-competitive mTOR inhibitors have already entered phase I and phase II trials, such as **AZD8055** (**R17**), **AZD2014** (**R18**), **MLN0128** (**R38**), **OSI-027** (**R39**), **CC-223** (**R44**) and **CC-115** (**R45**). As can be seen from Table 1, most of the inhibitors are used for the treatment of cancer and tumors, and there are also a few inhibitors for other diseases. **R24** shows anti-inflammatory effect. **R27**, **R28** and **R29** can be used for the treatment of epilepsy or other central nervous system diseases as well. **R36** has effect on virus-induced hand-foot-and-mouth disease. This will help people gain a more complete understanding of the applications for mTOR inhibitors.

### 3.3. Dual Binding Site Inhibitors

**Figure 8:**
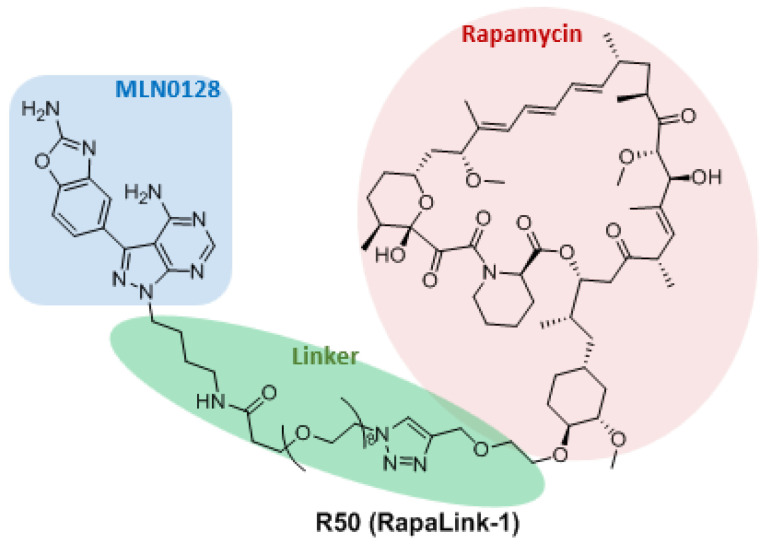
Structures of dual binding site inhibitor **RapaLink-1**.

Drug resistance is a problem that has plagued the development of effective mTOR inhibitors. Mutations leading to resistance to mTOR inhibitors occur in both treated and untreated patients. Rodrik-Outmezguine et al. proposed a new drug combination design strategy to overcome the resistance problem of existing mTOR inhibitors. After the ATP competitive inhibitor MLN0128 and rapamycin molecules were modified with polyethylene glycol, they were linked by azide-alkyne cycloaddition to obtain the compound **RapaLink-1** (**R50**, Figure 8), which has strong inhibitory effects on both rapamycin-binding site mutants and ATP-binding site mutants [93].

**Rapalink-1**, due to a unique structural arrangement and remarkable potential to inhibit mTOR became a leading structure in a new generation of mTOR inhibitors. Future development of RapaLinks will not be limited to link rapamycin and MLN0128. Both Rapalogs and ATP competitive inhibitors are alternative and there are many combinations to be studied. Members of both previous generations with remarkable pharmacokinetic profiles could be combined eventually to provide more potent inhibitors. The development of dual binding site mTOR inhibitors will provide a new clue for drug discovery.

### 3.4. Dual-Target mTOR Inhibitors

Long-term use of an mTOR inhibitor tends to lead to drug resistance, and targeting only a single pathway or target may lead to drug resistance through the bypass mechanisms. Therefore, we also summarize dual-target inhibitors, with a focus on dual-target inhibitors that act on multiple pathways.

#### 3.4.1. Dual mTOR/PI3K Inhibitors

**Figure 9:**
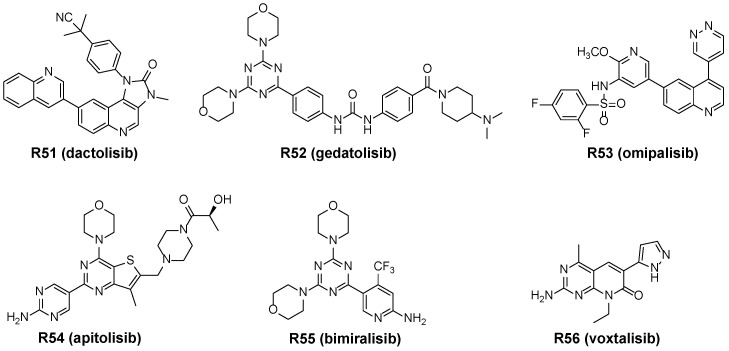
Structures of dual mTOR/PI3K inhibitors.

PI3K and mTOR both belong to the PIKK superfamily, and the p110 subunit of PI3K and catalytic domain of mTOR are structurally similar. Dual mTOR/PI3K inhibitors benefit from this structural similarity and inhibit mTORC1, mTORC2 and PI3K. At present, several dual mTOR/PI3K inhibitors have already entered phase I and phase II trials, such as dactolisib (NVP-BEZ235), gedatolisib (PKI-587), omipalisib (GSK2126458), apitolisib (GDC-0980), bimiralisib (PQR309), voxtalisib (XL765) (Figure 9).

Dactolisib (**R51**) is a pan-PI3K/mTOR dual inhibitor with IC_50_ values of 20.7 nM against mTOR and 4–75 nM against PI3K, respectively. **R51** inhibited the proliferation of tumor cells and was the first PI3K inhibitor to enter clinical trials [94]. 

Gedatolisib (**R52**) is an mTOR/PI3K dual inhibitor with a 2,4-dimorpholinyl-1,3,5-triazine structure, exhibits IC_50_ values of 1.6 nM and 0.4 nM for mTOR and PI3Kα, respectively. Compound **R52** inhibits the proliferation of BON, QGP-1, KRJI and LCC-18 cells [95,96]. Currently, gedatolisib is in Phase II clinical trials.

Omipalisib (**R53**) is an mTOR/PI3K dual inhibitor which has a pyridinesulfonamide structure with IC_50_ values of 0.3 nM for mTOR and 0.013 nM for PI3Kα. It inhibited the proliferation of HCC1954 and T-47D cells and tumor growth in a BT474 human tumor xenograft model [97]. At present, **R53** is in Phase I clinical trials for solid tumors and lymphomas.

Apitolisib (**R54**) is a pan-PI3K/mTOR inhibitor with Ki values of 17 nM against mTOR and 5–27 nM against PI3K, respectively. **R54** caused cell cycle arrest and induced apoptosis of tumor cells and displayed excellent inhibitory effect on different cancer cells such as breast cancer, pancreatic cancer, NSCLC and colon cancer cell lines [98]. Currently, apitolisib is in Phase I clinical trials.

Bimiralisib (**R55**) is a novel mTOR/PI3K dual inhibitor that shows IC_50_ values of 33–708 nM against PI3K and 89 nM against mTOR [99]. At present, **R55** is in Phase I/II trials for advanced solid tumors and refractory lymphoma [100].

Voxtalisib (**R56**) is an mTOR/PI3K dual inhibitor with a quinoxaline benzene sulfonamide structure, exhibits IC_50_ values of 150 nM and 1–43 nM for mTOR and PI3K, respectively. **R56** showed dose-dependent inhibition of phosphorylation of AKT, p70S6K, and S6 and significant reduction of tumor growth in multiple human xenograft models [101]. **R56** is in Phase II clinical trials for relapsed or refractory non-Hodgkin lymphoma and chronic lymphocytic leukemia [102].

#### 3.4.2. Dual mTOR/HDAC Inhibitors

**Figure 10:**
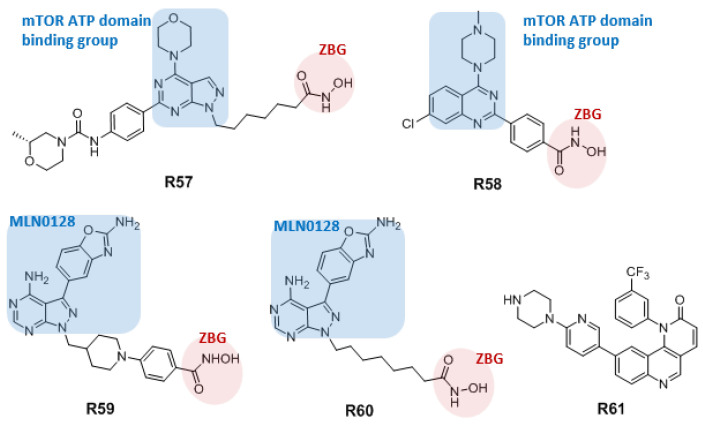
Structures of dual mTOR/HDAC inhibitors **R57**–**R60** and ATR/mTOR dual kinase inhibitor **R61**.

The abnormal expression or function of HDACs is associated with a variety of human malignancies, and HDAC inhibitors have been approved by the FDA for the treatment of hematological malignancies. Studies have found that the combined use of mTOR inhibitors and HDAC inhibitors can produce a synergistic effect that enhances the antitumor activity. On this basis, Chen et al. designed and synthesized a series of HDAC and mTOR dual-target inhibitors using a pyrimidine-pyrazolyl pharmacophore, of which **R57** (Figure 10) was the most active compound. It has strong inhibitory activity against mTOR and HDAC1 with IC_50_ values of 1.2 and 0.19 nM, respectively. Western blot analyses confirmed that treatment with **R57** leads to increased levels of H3 and α-tubulin acetylation and can downregulate the activity of proteins downstream of mTOR. Treatment with **R57** also leads to cell cycle arrest in G0/G1 and induces tumor cell apoptosis. This compound showed an antitumor activity comparable to a combination drug in the human myeloma cell MM1S xenograft tumor model, with a tumor growth inhibition rate of 72.5%, without causing significant weight loss or toxicity. These results suggest that **R57** may be a promising dual-target inhibitor for the treatment of hematological malignancies [103].

Yao et al. adopted a structure-based design strategy to shorten the connecting chain and reduce steric hindrance, and designed and synthesized a series of mTOR/HDAC6 dual-target inhibitors. Among them, the IC_50_ values of compound **R58** on mTOR and HDAC6 were 133.7 and 56 nM, respectively, and the compound inhibited the proliferation of the TNBC cell line MDA-MB-231. In addition, **R58** induced significant autophagy and apoptosis and inhibited the migration of MDA-MB-231 cells [104].

Zhang et al. connected the mTOR inhibitor **MLN0128** (**R38**) to the terminal zinc binding group (ZBG) of an HDAC inhibitor through a pharmacophore-merging strategy and discovered a novel mTOR/HDAC bifunctional inhibitor with pyrazolopyrimidine as the parent nucleus. The IC_50_ values of compound **R59** for mTOR and HDAC1 were 0.49 and 0.91 nM, respectively, and the inhibitory activity against MV4–11 cells (IC_50_ = 1.74 μM) was significantly better than those of the mTOR inhibitor **MLN0128** (IC_50_ = 5.84 μM) and the HDAC inhibitor **SAHA** (IC_50_ = 8.44 μM) [105].

Based on the mTOR inhibitors **MLN0128** (**R38**) and the HDAC inhibitor **SAHA**, Zhai et al. designed and synthesized novel hybrid molecules targeting mTOR and HDACs using a fusion-type molecular hybridization strategy. Among them, compound **R60** exhibited nanomolar inhibitory activity on mTOR kinase and HDACs, and antiproliferative activity on HepG2 cells, with IC_50_ value of 1.61 μM, which is comparable to **MLN0128** and **SAHA**. **R60** induces apoptosis by targeting mTOR and HDACs, blocks the HepG2 cell cycle in G0/G1, and inhibits cell migration [106].

#### 3.4.3. ATR/mTOR Dual Kinase Inhibitor

Human cells are continuously affected by various DNA-damaging endogenous stresses, including reactive oxygen species and replication errors, and exogenous stresses, including radiation and other environmental factors. DNA damage, if left unrepaired, can lead to deleterious mutations or induce abnormal cellular behavior, ultimately leading to the development of malignancies. ATR and ataxia telangiectasia mutated (ATM) kinases are major regulators of the DNA damage response signaling pathway. Inhibiting ATR helps to preferentially kill cancer cells that continue to replicate. **Torin2** (**R31**) was found to inhibit ATR but with low selectivity. Bhakuni et al., therefore, designed and synthesized **Torin2** analogs, and through preliminary screening, they obtained the compound **R61** (Figure 10) with significant anticancer activity on HCT 116 cells. Compound **R61** inhibited both ATR and mTOR kinases more strongly than it inhibited ATM. **R61** exhibited better specific inhibition of ATR at lower concentrations than did **Torin2**, and the half-life of **R61** (t_1/2_ = 157 min) was increased relative to that of **Torin2**, while the clearance rate in mice was also increased [107].

The concept of dual-target drug/inhibition refers to a single molecule that interacts with two different target proteins. This dual inhibition can be vertical (two target proteins on the same pathway, such as dual mTOR/PI3K inhibitors) or horizontal (two target proteins on different pathways, such as dual mTOR/HDAC inhibitors and ATR/mTOR dual kinase inhibitor), that results in synergistic efficacy. The dual-target inhibitors could increase or maintain the desired therapeutic efficacy potentially requiring a lower dose, and minimize drug resistance compared to single-target treatment. Dual mTOR/PI3K inhibitors are mostly obtained from low-activity inhibitors by structural optimization to improve activity. Indeed, earlier developed PI3K inhibitors also showed some mTOR inhibition. Dual mTOR/HDAC inhibitors are obtained based upon defined pharmacophores for mTOR inhibitors and HDAC inhibitors. Structurally, dual mTOR/HDAC inhibitors consist of two functional components, one end is an mTOR ATP domain binding group, and the other end is a zinc binding group of HDAC inhibitor, and the two are connected by a linker. ATR/mTOR dual kinase inhibitor **R61** is optimized to improve ATR inhibitory activity on the basis of an mTOR inhibitor.

## 4. Summary and Prospects

The mTOR pathway affects many cellular functions, plays a crucial role in cell growth and proliferation, and is involved in physiological processes that are closely related to health, disease, and aging. As a key kinase regulating the metabolic pathways in cells, the dysregulation of signaling through mTOR occurs in a variety of human diseases, including cancer, obesity, type II diabetes, immune diseases, and neurodegenerative diseases. This review summarized three classes of inhibitors targeting mTOR and also described dual-target mTOR inhibitors.

Among the allosteric inhibitors of mTOR, rapamycin and its derivatives were the earliest mTOR inhibitors used in the treatment of immune diseases and tumors. Their incomplete inhibition of mTOR and the existence of negative feedback pathways weakens the inhibitory effect on mTOR. Such inhibitors have complex structures, large molecular weights, and many chiral structures, and are therefore difficult to synthesize. The recent discovery of non-rapalog allosteric inhibitors developed based on an in silico and in cell hybrid strategy will open up new directions for the development of mTOR allosteric inhibitors.

ATP-competitive mTOR inhibitors directly act on the ATP-binding site of the catalytic domain of mTOR kinase, and can inhibit both mTORC1 and mTORC2, leading to greater therapeutic potential. With the discovery of ATP-competitive inhibitors of many novel structural frameworks, ATP competitive inhibitors have become a focus of mTOR inhibitor research. Moreover, the current ATP-competitive inhibitors are not limited to the treatment of tumors and cancers, but also have applications in central nervous system diseases, viral infections, inflammation, and other disorders, which lay the foundation for expanding the indications of mTOR inhibitors.

Long-term use of the same mTOR inhibitor is prone to drug resistance, and targeting only a single pathway may also lead to drug resistance through the bypass mechanisms. Whether it is a dual-binding site inhibitor obtained by a drug combination design strategy or a dual-target inhibitor obtained by a strategy such as pharmacophore splicing, multiple targeting would be beneficial to the solving of the drug resistance problem. In order to overcome mTOR inhibitor resistance, more novel drug design strategies should be tried, such as designing proteolysis targeting chimera molecules targeting mTOR or multi-target inhibitors.

In conclusion, the work described above will provide new avenues for the development of mTOR inhibitors, as well as provide the basis for basic research and clinical treatment of human diseases.

## Figures and Tables

**Table 1:** Summarized study types and applications of mTOR inhibitors.

Inhibitors	Type of Study	Inhibitors’ Applications
**R1**	clinical use	lymphangioleiomyomatosis
**R2**	clinical use	advanced renal cell carcinoma (RCC)
**R3**	clinical use	advanced RCC, subependymal giant cell astrocytoma, and tuberous sclerosis complex (TSC)
**R4**	clinical studies	osteosarcoma
**R5**	animal studies	breast cancer
**R6**	cells	breast cancer, glioma
**R7**	cells	breast cancer, glioma
**R8**	cells	breast cancer, glioma
**R9**	animal studies	prostate cancer, glioma
**R10**	animal studies	ovarian cancer, colon cancer
**R11**	animal studies	ovarian cancer, colon cancer
**R12**	animal studies	ovarian cancer, prostate cancer
**R13**	animal studies	Non-Hodgkin’s lymphoma, solid tumor
**R14**	animal studies	prostate tumor
**R15**	animal studies	prostate cancer, breast cancer
**R16**	animal studies	breast cancer
**R17**	clinical studies	hepatocellular carcinoma (HCC)
**R18**	clinical studies	colorectal cancer, advanced RCC
**R19**	cells	breast cancer, bladder cancer
**R20**	cells	prostate cancer, breast cancer
**R21**	animal studies	glioma
**R22**	cells	breast cancer
**R23**	animal studies	breast cancer, endometrial cancer
**R24**	animal studies	inflammation
**R25**	animal studies	systemic tumor
**R26**	kinase assays	cancer
**R27**	animal studies	ovarian cancer, chronic epilepsy
**R28**	animal studies	TSC-induced epilepsy, CNS disorders
**R29**	animal studies	TSC-induced epilepsy, CNS disorders
**R30**	animal studies	glioblastoma tumor
**R31**	animal studies	papillary thyroid carcinoma
**R32**	cells	ovarian cancer, colon cancer
**R33**	cells	prostate cancer, colon cancer, breast cancer
**R34**	cells	acute promyelocytic leukemia, pancreatic cancer, liver cancer, prostate cancer, colon cancer
**R35**	animal studies	breast cancer, cervical cancer
**R36**	cells	hand-foot-and-mouth disease
**R37**	animal studies	bladder cancer
**R38**	clinical studies	TS, acute lymphoblastic leukemia
**R39**	clinical studies	colon cancer, breast cancer
**R40**	cells	rhabdomyosarcoma
**R41**	animal studies	breast cancer
**R42**	animal studies	endometrial carcinoma, esophageal carcinoma
**R43**	animal studies	prostatic cancer
**R44**	clinical studies	diffuse large B cell lymphoma, breast cancer, lung cancer
**R45**	clinical studies	breast cancer, lung cancer
**R46**	cells	ovarian cancer, prostate cancer
**R47**	animal studies	triple-negative breast cancer (TNBC)
**R48**	cells	TNBC, breast cancer
**R49**	animal studies	prostate cancer, endometrial cancer
**R50**	animal studies	breast cancer
**R51**	clinical studies	bladder cancer, pancreatic cancer, breast cancer, renal cell carcinoma, solid tumor, prostate cancer
**R52**	clinical studies	metastatic breast cancer, pancreatic cancer, colon cancer
**R53**	clinical studies	solid tumor, lymphoma
**R54**	clinical studies	breast cancer, pancreatic cancer, non-small cell lung cancer, colon cancer
**R55**	clinical studies	advanced solid tumor, refractory lymphoma
**R56**	clinical studies	relapsed or refractory non-Hodgkin’s lymphoma, chronic lymphocytic leukemia
**R57**	cells	leukemia, myeloma
**R58**	cells	TNBC
**R59**	cells	monocytic leukemia, prostate cancer, colon cancer
**R60**	cells	liver cancer, breast cancer, solid tumor, prostate cancer
**R61**	animal studies	carcinoma of colon

## Data Availability

Not applicable.

## Abbreviations

AKT	protein kinase-B
AMBRA1	autophagy/beclin 1 regulator 1
AMP	adenosine monophosphate
AMPK	adenosine monophosphate-activated protein kinase
ATG	autophagy-related gene
ATM	ataxia telangiectasia mutated
ATP	adenosine triphosphate
ATR	ataxia telangiectasia mutated and Rad-3 related
eIF4E	eukaryotic translation initiation factor 4E
EV71	enterovirus 71
FAT	focal adhesion targeting domain
FATC	focal adhesion targeting domain at the C-terminus
FDA	Food and Drug Administration
FKBP12	FK506-binding protein of 12 kD
FRB	FKBP12-rapamycin binding domain
GDP	guanosine diphosphate
GEF	guanylate exchange factor
GTP	guanosine triphosphate
HDACs	histone deacetylases
hERG	human ether-a-go-go related gene
HFMD	hand-foot-and-mouth disease
HGMCS	3-hydroxy 3-methyl glutaryl CoA (HMGCoA) synthase
HMGCoA	3-hydroxy 3-methyl glutaryl CoA
KIN	kinase domain
MEK	mitogen-activated protein kinase kinase
mLST8	mammalian lethal with sec-13 protein 8
mSIN1	mammalian stress-activated map kinase-interacting protein 1
mTOR	mammalian target of rapamycin
mTORC	mTOR complex
mTOR^ΔN^	mTOR N-terminal truncation mutant
NRD	negative regulatory domain
PI3K	phosphoinositide 3-kinase
PIKK	phosphatidylinositol-3-kinase-related kinase
PIP_2_	phosphatidylinositol (4,5)-biphosphate
PIP_3_	phosphatidylinositol (3,4,5)-triphosphate
PKCα	protein kinase Cα
PRAS40	prolin-rich Akt substrate of 40 kD
REDD1	regulated in development and DNA damage responses-1
Rheb	ras homolog enriched in brain 1
S6K1	ribosomal protein S6 kinase 1
SGK1	serum- and glucocorticoid-induced protein kinase 1
SUPT6H	suppressor of Ty homologue-6
TFEB	transcription factor EB
TNBC	triple-negative breast cancer
TSC	tuberous sclerosis complex
ULK	unc-51-like kinase
VPS34	vacuolar protein sorting 34
4E-BP1	eukaryotic translation initiation factor 4E-binding protein 1
5′ TOP	5′ terminal oligopyrimidine tract mRNA translation protein

## References

[1] Laplante M., Sabatini D.M. (2012). mTOR signaling in growth control and disease. Cell.

[2] Keith C.T., Schreiber S.L. (1995). PIK-related kinases: DNA repair, recombination, and cell cycle checkpoints. Science.

[3] Jacinto E., Loewith R., Schmidt A., Lin S., Ruegg M.A., Hall A., Hall M.N. (2004). Mammalian TOR complex 2 controls the actin cytoskeleton and is rapamycin insensitive. Nat. Cell Biol..

[4] Chiarini F., Evangelisti C., McCubrey J.A., Martelli A.M. (2015). Current treatment strategies for inhibiting mTOR in cancer. Trends Pharmacol. Sci..

[5] Lv X., Ma X., Hu Y. (2013). Furthering the design and the discovery of small molecule ATP-competitive mTOR inhibitors as an effective cancer treatment. Expert Opin. Drug Discov..

[6] Gingras A.C., Raught B., Sonenberg N. (2001). Regulation of translation initiation by FRAP/mTOR. Genes Dev..

[7] Sekulic A., Hudson C.C., Homme J.L., Yin P., Otterness D.M., Karnitz L.M., Abraham R.T. (2000). A direct linkage between the phosphoinositide 3-kinase-AKT signaling pathway and the mammalian target of rapamycin in mitogen-stimulated and transformed cells. Cancer Res..

[8] Kallen J.A., Sedrani R., Cottens S. (1996). X-ray crystal structure of 28-O-methylrapamycin complexed with FKBP12: Is the cyclohexyl moiety part of the effector domain of rapamycin?. J. Am. Chem. Soc..

[9] Choi J., Chen J., Schreiber S.L., Clardy J. (1996). Structure of the FKBP12-rapamycin complex interacting with the binding domain of human FRAP. Science.

[10] Yang H., Rudge D.G., Koos J.D., Vaidialingam B., Yang H.J., Pavletich N.P. (2013). mTOR kinase structure, mechanism and regulation. Nature.

[11] Kim D.H., Sarbassov D.D., Ali S.M., Latek R.R., Guntur K.V., Erdjument-Bromage H., Tempst P., Sabatini D.M. (2003). GβL, a positive regulator of the rapamycin-sensitive pathway required for the nutrient-sensitive interaction between raptor and mTOR. Mol. Cell.

[12] Peterson T.R., Laplante M., Thoreen C.C., Sancak Y., Kang S.A., Kuehl W.M., Gray N.S., Sabatini D.M. (2009). DEPTOR is an mTOR inhibitor frequently overexpressed in multiple myeloma cells and required for their survival. Cell.

[13] Sancak Y., Thoreen C.C., Peterson T.R., Lindquist R.A., Kang S.A., Spooner E., Carr S.A., Sabatini D.M. (2007). PRAS40 is an insulin-regulated inhibitor of the mTORC1 protein kinase. Mol. Cell.

[14] Xu K., Liu P., Wei W. (2014). mTOR signaling in tumorigenesis. Biochim. Biophys. Acta.

[15] Vignot S., Faivre S., Aguirre D., Raymond E. (2005). mTOR-targeted therapy of cancer with rapamycin derivatives. Ann. Oncol..

[16] Qian D.C., Xiao X., Byun J., Suriawinata A.A., Her S.C., Amos C.I., Barth R.J. (2017). PI3K/Akt/mTOR signaling and plasma membrane proteins are implicated in responsiveness to adjuvant dendritic cell vaccination for metastatic colorectal cancer. Clin. Cancer Res..

[17] Lee M., Minaskan N., Wiedemann T., Irmler M., Beckers J., Yousefi B.H., Kaissis G., Braren R., Laitinen I., Pellegata N.S. (2017). Targeting PI3K/mTOR signaling exerts potent antitumor activity in pheochromocytoma in vivo. Endocr. Relat. Cancer..

[18] Li X., Wu C., Chen N., Gu H., Yen A., Cao L., Wang E., Wang L. (2016). PI3K/Akt/mTOR signaling pathway and targeted therapy for glioblastoma. Oncotarget.

[19] Yu Y., Hou L., Song H., Xu P., Sun Y., Wu K. (2017). Akt/AMPK/mTOR pathway was involved in the autophagy induced by vitamin E succinate in human gastric cancer SGC-7901 cells. Mol. Cell. Biochem..

[20] Zheng X.T., Wu Z.H., Wei Y., Dai J.J., Yu G.F., Yuan F., Ye L.C. (2017). Induction of autophagy by salidroside through the AMPK-mTOR pathway protects vascular endothelial cells from oxidative stress-induced apoptosis. Mol. Cell. Biochem..

[21] Duan P., Hu C., Quan C., Yu T., Huang W., Chen W., Tang S., Shi Y., Martin F.L., Yang K. (2017). 4-Nonylphenol induces autophagy and attenuates mTOR-p70S6K/4EBP1 signaling by modulating AMPK activation in Sertoli cells. Toxicol. Lett..

[22] Brugarolas J., Lei K., Hurley R.L., Manning B.D., Reiling J.H., Hafen E., Witters L.A., Ellisen L.W., Kaelin W.G. (2004). Regulation of mTOR function in response to hypoxia by REDD1 and the TSC1/TSC2 tumor suppressor complex. Genes Dev..

[23] DeYoung M.P., Horak P., Sofer A., Sgroi D., Ellisen L.W. (2008). Hypoxia regulates TSC1/2-mTOR signaling and tumor suppression through REDD1-mediated 14-3-3 shuttling. Genes Dev..

[24] Feng Z., Zhang H., Levine A.J., Jin S. (2005). The coordinate regulation of the p53 and mTOR pathways in cells. Proc. Natl. Acad. Sci. USA.

[25] Stambolic V., MacPherson D., Sas D., Lin Y., Snow B., Jang Y., Benchimol S., Mak T.W. (2001). Regulation of PTEN transcription by p53. Mol. Cell.

[26] Jewell J.L., Russell R.C., Guan K.L. (2013). Amino acid signalling upstream of mTOR. Nat. Rev. Mol. Cell Biol..

[27] Jewell J.L., Kim Y.C., Russell R.C., Yu F.X., Park H.W., Plouffe S.W., Tagliabracci V.S., Guan K.L. (2015). Differential regulation of mTORC1 by leucine and glutamine. Science.

[28] Wang S., Tsun Z.Y., Wolfson R.L., Shen K., Wyant G.A., Plovanich M.E., Yuan E.D., Jones T.D., Chantranupong L., Comb W. (2015). Metabolism. Lysosomal amino acid transporter SLC38A9 signals arginine sufficiency to mTORC1. Science.

[29] Varea O., Escoll M., Diez H., Garrido J.J., Wandosell F. (2013). Oestradiol signalling through the Akt-mTORC1-S6K1. Biochim. Biophys. Acta.

[30] Ben-Hur V., Denichenko P., Siegfried Z., Maimon A., Krainer A., Davidson B., Karni R. (2013). S6K1 alternative splicing modulates its oncogenic activity and regulates mTORC1. Cell Rep..

[31] Siroky B.J., Bitzer M. (2009). The growing importance of mTORC1-S6K1 signaling in kidney. Am. J. Physiol. Renal. Physiol..

[32] Josse L., Xie J., Proud C.G., Smales C.M. (2016). mTORC1 signalling and eIF4E/4E-BP1 translation initiation factor stoichiometry influence recombinant protein productivity from GS-CHOK1 cells. Biochem. J..

[33] Livingstone M., Bidinosti M. (2012). Rapamycin-insensitive mTORC1 activity controls eIF4E:4E-BP1 binding. F1000Res..

[34] Zhang D., Contu R., Latronico M.V., Zhang J., Rizzi R., Catalucci D., Miyamoto S., Huang K., Ceci M., Gu Y. (2010). MTORC1 regulates cardiac function and myocyte survival through 4E-BP1 inhibition in mice. J. Clin. Investig..

[35] Kim Y.C., Guan K.L. (2015). mTOR: A pharmacologic target for autophagy regulation. J. Clin. Investig..

[36] White E. (2015). The role for autophagy in cancer. J. Clin. Investig..

[37] Zhao J., Zhai B., Gygi S.P., Goldberg A.L. (2015). mTOR inhibition activates overall protein degradation by the ubiquitin proteasome system as well as by autophagy. Proc. Natl. Acad. Sci. USA.

[38] Sarbassov D.D., Ali S.M., Kim D.H., Guertin D.A., Latek R.R., Erdjument-Bromage H., Tempst H.P., Sabatini D.M. (2004). Rictor, a novel binding partner of mTOR, defines a rapamycin-insensitive and raptor-independent pathway that regulates the cytoskeleton. Curr. Biol..

[39] Pearce L.R., Sommer E.M., Sakamoto K., Wullschleger S., Alessi D.R. (2011). Protor-1 is required for efficient mTORC2-mediated activation of SGK1 in the kidney. Biochem. J..

[40] Sarbassov D.D., Guertin D.A., Ali S.M., Sabatini D.M. (2005). Phosphorylation and regulation of Akt/PKB by the rictor-mTOR complex. Science.

[41] Garcia-Martinez J.M., Alessi D.R. (2008). mTOR complex 2 (mTORC2) controls hydrophobic motif phosphorylation and activation of serum- and glucocorticoid-induced protein kinase 1 (SGK1). Biochem. J..

[42] Liu Q., Thoreen C., Wang J., Sabatini D., Gray N.S. (2009). mTOR mediated anti-cancer drug discovery. Drug Discov. Today Ther. Strateg..

[43] Wander S.A., Hennessy B.T., Slingerland J.M. (2011). Next-generation mTOR inhibitors in clinical oncology: How pathway complexity informs therapeutic strategy. J. Clin. Investig..

[44] Baselga J., Campone M., Piccart M., Burris H.A., Rugo H.S., Sahmoud T., Noguchi S., Gnant M., Pritchard K.I., Lebrun F. (2012). Everolimus in postmenopausal hormone-receptor-positive advanced breast cancer. N. Engl. J. Med..

[45] Mita M.M., Gong J., Chawla S.P. (2013). Ridaforolimus in advanced or metastatic soft tissue and bone sarcomas. Expert Rev. Clin. Pharmacol..

[46] Shams R., Matsukawa A., Ochi Y., Ito Y., Miyatake H. (2022). In Silico and In Cell Hybrid Selection of Nonrapalog Ligands to Allosterically Inhibit the Kinase Activity of mTORC1. J. Med. Chem..

[47] Yu K., Toral-Barza L., Shi C., Zhang W.G., Lucas J., Shor B., Kim J., Verheijen J., Curran K., Malwitz D.J. (2009). Biochemical, cellular, and in vivo activity of novel ATP-competitive and selective inhibitors of the mammalian target of rapamycin. Cancer. Res..

[48] Cohen F., Bergeron P., Blackwood E., Bowman K.K., Chen H., Dipasquale A.G., Epler J.A., Koehler M.F., Lau K., Lewis C. (2011). Potent, selective, and orally bioavailable inhibitors of mammalian target of rapamycin (mTOR) kinase based on a quaternary substituted dihydrofuropyrimidine. J. Med. Chem..

[49] Koehler M.F., Bergeron P., Blackwood E., Bowman K.K., Chen Y.H., Deshmukh G., Ding X., Epler J., Lau K., Lee L. (2012). Potent, selective, and orally bioavailable inhibitors of the mammalian target of rapamycin kinase domain exhibiting single agent antiproliferative activity. J. Med. Chem..

[50] Pei Z., Blackwood E., Liu L., Malek S., Belvin M., Koehler M.F., Ortwine D.F., Chen H., Cohen F., Kenny J.R. (2013). Discovery and biological profiling of potent and selective mTOR inhibitor GDC-0349. ACS Med. Chem. Lett..

[51] Estrada A.A., Shore D.G., Blackwood E., Chen Y.H., Deshmukh G., Ding X., Dipasquale A.G., Epler J.A., Friedman L.S., Koehler M.F. (2013). Pyrimidoaminotropanes as potent, selective, and efficacious small molecule kinase inhibitors of the mammalian target of rapamycin (mTOR). J. Med. Chem..

[52] Liu K.K., Bailey S., Dinh D.M., Lam H., Li C., Wells P.A., Yin M.J., Zou A. (2012). Conformationally-restricted cyclic sulfones as potent and selective mTOR kinase inhibitors. Bioorg. Med. Chem. Lett..

[53] Malagu K., Duggan H., Menear K., Hummersone M., Gomez S., Bailey C., Edwards P., Drzewiecki J., Leroux F., Quesada M.J. (2009). The discovery and optimisation of pyrido[2,3-*d*]pyrimidine-2,4-diamines as potent and selective inhibitors of mTOR kinase. Bioorg. Med. Chem. Lett..

[54] Pike K.G., Malagu K., Hummersone M.G., Menear K.A., Duggan H.M., Gomez S., Martin N.M., Ruston L., Pass S.L., Pass M. (2013). Optimization of potent and selective dual mTORC1 and mTORC2 inhibitors: The discovery of AZD8055 and AZD2014. Bioorg. Med. Chem. Lett..

[55] Huang S., Yang Z.J., Yu C., Sinicrope F.A. (2011). Inhibition of mTOR kinase by AZD8055 can antagonize chemotherapy-induced cell death through autophagy induction and down-regulation of p62/sequestosome 1. J. Biol. Chem..

[56] Holt S.V., Logie A., Davies B.R., Alferez D., Runswick S., Fenton S., Chresta C.M., Gu Y., Zhang J., Wu Y.L. (2012). Enhanced apoptosis and tumor growth suppression elicited by combination of MEK (selumetinib) and mTOR kinase inhibitors (AZD8055). Cancer Res..

[57] Marshall G., Howard Z., Dry J., Fenton S., Heathcote D., Gray N., Keen H., Logie A., Holt S., Smith P. (2011). Benefits of mTOR kinase targeting in oncology: Pre-clinical evidence with AZD8055. Biochem. Soc. Trans..

[58] Lee W., Ortwine D.F., Bergeron P., Lau K., Lin L., Malek S., Nonomiya. J., Pei Z., Robarge K.D., Schmidt S. (2013). A hit to lead discovery of novel *N*-methylated imidazolo-, pyrrolo-, and pyrazolo-pyrimidines as potent and selective mTOR inhibitors. Bioorg. Med. Chem. Lett..

[59] Finlay M.R., Buttar D., Critchlow S.E., Dishington A.P., Fillery S.M., Fisher E., Glossop S.C., Graham M.A., Johnson T., Lamont G.M. (2012). Sulfonyl-morpholino-pyrimidines: SAR and development of a novel class of selective mTOR kinase inhibitor. Bioorg. Med. Chem. Lett..

[60] Pike K.G., Morris J., Ruston L., Pass S.L., Greenwood R., Williams E.J., Demeritt J., Culshaw J.D., Gill K., Pass M. (2015). Discovery of AZD3147: A potent, selective dual inhibitor of mTORC1 and mTORC2. J. Med. Chem..

[61] Zhu W., Sun C., Xu S., Wu C., Wu J., Xu M., Zhao H., Chen L., Zeng W., Zheng P. (2014). Design, synthesis, anticancer activity and docking studies of novel 4-morpholino-7,8-dihydro-5*H*-thiopyrano[4,3-*d*]pyrimidine derivatives as mTOR inhibitors. Bioorg. Med. Chem..

[62] Mao B., Gao S., Weng Y., Zhang L., Zhang L. (2017). Design, synthesis, and biological evaluation of imidazo[1,2-*b*]pyridazine derivatives as mTOR inhibitors. Eur. J. Med. Chem..

[63] Cansfield A.D., Ladduwahetty T., Sunose M., Ellard K., Lynch R., Newton A.L., Lewis A., Bennett G., Zinn N., Thomson D.W. (2016). CZ415, a highly selective mTOR inhibitor showing in vivo efficacy in a collagen induced arthritis model. ACS Med. Chem. Lett..

[64] Borsari C., Rageot D., Dall’Asen A., Bohnacker T., Melone A., Sele A.M., Jackson E., Langlois J.B., Beaufils F., Hebeisen P. (2019). A Conformational Restriction Strategy for the Identification of a Highly Selective Pyrimido-pyrrolo-oxazine mTOR Inhibitor. J. Med. Chem..

[65] Hobbs H., Bravi G., Campbell I., Convery M., Davies H., Inglis G., Pal S., Peace S., Redmond J., Summers D. (2019). Discovery of 3-oxabicyclo[4.1.0]heptane, a non-nitrogen containing morpholine isostere, and its application in novel inhibitors of the PI3K-AKT-mTOR pathway. J. Med. Chem..

[66] Rageot D., Bohnacker T., Melone A., Langlois J.B., Borsari C., Hillmann P., Sele A.M., Beaufils F., Zvelebil M., Hebeisen P. (2018). Discovery and preclinical characterization of 5-[4,6-bis({3-oxa-8-azabicyclo[3.2.1]octan-8-yl)-1,3,5-triazin-2-yl]-4-(difluoromethyl)pyridin-2-amine (PQR620), a highly potent and selective mtorc1/2 inhibitor for cancer and neurological disorders. J. Med. Chem..

[67] Borsari C., Keles E., Rageot D., Treyer A., Bohnacker T., Bissegger L., De Pascale M., Melone A., Sriramaratnam R., Beaufils F. (2020). 4-(Difluoromethyl)-5-(4-((3R,5S)-3,5-dimethylmorpholino)-6-((R)-3-methylmorpholino)-1,3,5-triazin-2-yl)pyridin-2-amine (PQR626), a Potent, Orally Available, and Brain-Penetrant mTOR Inhibitor for the Treatment of Neurological Disorders. J. Med. Chem..

[68] Bonazzi S., Goold C.P., Gray A., Thomsen N.M., Nunez J., Karki R.G., Gorde A., Biag J.D., Malik H.A., Sun Y. (2020). Discovery of a Brain-Penetrant ATP-Competitive Inhibitor of the Mechanistic Target of Rapamycin (mTOR) for CNS Disorders. J. Med. Chem..

[69] Liu Q., Chang J.W., Wang J., Kang S.A., Thoreen C.C., Markhard A., Hur W., Zhang J., Sim T., Sabatini D.M. (2010). Discovery of 1-(4-(4-propionylpiperazin-1-yl)-3-(trifluoromethyl)phenyl)-9-(quinolin-3-yl)benzo[*h*][1,6]naphthyridin-2(1*H*)-one as a highly potent, selective mammalian target of rapamycin (mTOR) inhibitor for the treatment of cancer. J. Med. Chem..

[70] Thoreen C.C., Kang S.A., Chang J.W., Liu Q., Zhang J., Gao Y., Reichling L.J., Sim T., Sabatini D.M., Gray N.S. (2009). An ATP-competitive mammalian target of rapamycin inhibitor reveals rapamycin-resistant functions of mTORC1. J. Biol. Chem..

[71] Liu Q., Wang J., Kang S.A., Thoreen C.C., Hur W., Ahmed T., Sabatini D.M., Gray N.S. (2011). Discovery of 9-(6-aminopyridin-3-yl)-1-(3-(trifluoromethyl)phenyl)benzo[*H*][1,6]naphthyridin-2(1*H*)-one (Torin2) as a potent, selective, and orally available mammalian target of rapamycin (mTOR) inhibitor for treatment of cancer. J. Med. Chem..

[72] Venkateswarlu V., Pathania A.S., Kumar K.A.A., Mahajan P., Nargotra A., Vishwakarma R.A., Malik F.A., Sawant S.D. (2015). 4-(*N*-Phenyl-*N′*-substituted benzenesulfonyl)-6-(4-hydroxyphenyl)quinolines as inhibitors of mammalian target of rapamycin. Bioorg. Med. Chem..

[73] Ma X., Lv X., Qiu N., Yang B., He Q., Hu Y. (2015). Discovery of novel quinoline-based mTOR inhibitors via introducing intra-molecular hydrogen bonding scaffold (iMHBS): The design, synthesis and biological evaluation. Bioorg. Med. Chem..

[74] Miyanaga A., Janso J.E., McDonald L., He M., Liu H., Barbieri L., Eustáquio A.S., Fielding E.N., Carter G.T., Jensen P.R. (2011). Discovery and assembly-line biosynthesis of the lymphostin pyrroloquinoline alkaloid family of mTOR inhibitors in Salinispora bacteria. J. Am. Chem. Soc..

[75] Guo Q., Yu C., Zhang C., Li Y., Wang T., Huang Z., Wang X., Zhou W., Li Y., Qin Z. (2018). Highly Selective, Potent, and Oral mTOR Inhibitor for Treatment of Cancer as Autophagy Inducer. J. Med. Chem..

[76] Hao T., Li Y., Fan S., Li W., Wang S., Li S., Cao R., Zhong W. (2019). Design, synthesis and pharmacological evaluation of a novel mTOR-targeted anti-EV71 agent. Eur. J. Med. Chem..

[77] Apsel B., Blair J.A., Gonzalez B., Nazif T.M., Feldman M.E., Aizenstein B., Hoffman R., Williams R.L., Shokat K.M., Knight Z.A. (2008). Targeted polypharmacology: Discovery of dual inhibitors of tyrosine and phosphoinositide kinases. Nat. Chem. Biol..

[78] Hayman T.J., Wahba A., Rath B.H., Bae H., Kramp T., Shankavaram U.T., Camphausen K., Tofilon P.J. (2014). The ATP-competitive mTOR inhibitor INK128 enhances in vitro and in vivo radiosensitivity of pancreatic carcinoma cells. Clin. Cancer Res..

[79] Guo Y., Kwiatkowski D.J. (2013). Equivalent benefit of rapamycin and a potent mTOR ATP-competitive inhibitor, MLN0128 (INK128), in a mouse model of tuberous sclerosis. Mol. Cancer Res..

[80] Janes M.R., Vu C., Mallya S., Shieh M.P., Limon J.J., Li L.S., Jessen K.A., Martin M.B., Ren P., Lilly M.B. (2013). Efficacy of the investigational mTOR kinase inhibitor MLN0128/INK128 in models of B-cell acute lymphoblastic leukemia. Leukemia.

[81] Crew A.P., Bhagwat S.V., Dong H., Bittner M.A., Chan A., Chen X., Coate H., Cooke A., Gokhale P.C., Honda A. (2011). Imidazo[1,5-*a*]pyrazines: Orally efficacious inhibitors of mTORC1 and mTORC2. Bioorg. Med. Chem. Lett..

[82] Bhagwat S.V., Gokhale P.C., Crew A.P., Cooke A., Yao Y., Mantis C., Kahler J., Workman J., Bittner M., Dudkin L. (2011). Preclinical characterization of OSI-027, a potent and selective inhibitor of mTORC1 and mTORC2: Distinct from rapamycin. Mol. Cancer Ther..

[83] Bhagwat S.V., Crew A.P. (2010). Novel inhibitors of mTORC1 and mTORC2. Curr. Opin. Investig. Drugs..

[84] Takeuchi C.S., Kim B.G., Blazey C.M., Ma S., Johnson H.W., Anand N.K., Arcalas A., Baik T.G., Buhr C.A., Cannoy J. (2013). Discovery of a novel class of highly potent, selective, ATP-competitive, and orally bioavailable inhibitors of the mammalian target of rapamycin (mTOR). J. Med. Chem..

[85] Peterson E.A., Boezio A.A., Andrews P.S., Boezio C.M., Bush T.L., Cheng A.C., Choquette D., Coats J.R., Colletti A.E., Copeland K.W. (2012). Discovery and optimization of potent and selective imidazopyridine and imidazopyridazine mTOR inhibitors. Bioorg. Med. Chem. Lett..

[86] Mortensen D.S., Sapienza J., Lee B.G., Perrin-Ninkovic S.M., Harri R., Shevlin G., Parnes J.S., Whitefield B., Hickman M., Khambatta G. (2013). Use of core modification in the discovery of CC214-2, an orally available, selective inhibitor of mTOR kinase. Bioorg. Med. Chem. Lett..

[87] Mortensen D.S., Perrin-Ninkovic S.M., Shevlin G., Zhao J., Packard G., Bahmanyar S., Correa M., Elsner J., Harris R., Lee B.G. (2015). Discovery of mammalian target of rapamycin (mTOR) kinase inhibitor CC-223. J. Med. Chem..

[88] Mortensen D.S., Perrin-Ninkovic S.M., Shevlin G., Elsner J., Zhao J., Whitefield B., Tehrani L., Sapienza J., Riggs J.R., Parnes J.S. (2015). Optimization of a series of triazole containing mammalian target of rapamycin (mTOR) kinase inhibitors and the discovery of CC-115. J. Med. Chem..

[89] Reddy G.L., Guru S.K., Srinivas M., Pathania A.S., Mahajan P., Nargotra A., Bhushan S., Vishwakarma R.A., Sawant S.D. (2014). Synthesis of 5-substituted-1H-pyrazolo[4,3-d]pyrimidin-7(6H)-one analogs and their biological evaluation as anticancer agents: mTOR inhibitors. Eur. J. Med. Chem..

[90] Fouque A., Delalande O., Jean M., Castellano R., Josselin E., Malleter M., Shoji K.F., Hung M.D., Rampanarivo H., Collette Y. (2015). A novel covalent mTOR inhibitor, DHM25, shows in vivo antitumor activity against triple-negative breast cancer cells. J. Med. Chem..

[91] Xu T., Zhang J., Yang C., Pluta R., Wang G., Ye T., Ouyang L. (2021). Identification and optimization of 3-bromo-N’-(4-hydroxybenzylidene)-4-methylbenzohydrazide derivatives as mTOR inhibitors that induce autophagic cell death and apoptosis in triple-negative breast cancer. Eur. J. Med. Chem..

[92] Jin S., Mikami S., Scorah N., Chen Y., Halkowycz P., Shi L., Kahana J., Vincent P., de Jong R., Atienza J. (2019). Rational discovery of a highly novel and selective mTOR inhibitor. Bioorg. Med. Chem. Lett..

[93] Rodrik-Outmezguine V.S., Okaniwa M., Yao Z., Novotny C.J., McWhirter C., Banaji A., Won H., Wong W., Berger M., de Stanchina E. (2016). Overcoming mTOR resistance mutations with a new-generation mTOR inhibitor. Nature.

[94] Serra V., Markman B., Scaltriti M., Eichhorn P.J., Valero V., Guzman M., Botero M.L., Llonch E., Atzori F., Di Cosimo S. (2008). NVP-BEZ235, a dual PI3K/mTOR inhibitor, prevents PI3K signaling and inhibits the growth of cancer cells with activating PI3K mutations. Cancer Res..

[95] Venkatesan A.M., Dehnhardt C.M., Santos E.D., Chen Z., Santos O.D., Ayral-Kaloustian S., Khafizova G., Brooijmans N., Mallon R., Hollander I. (2010). Bis(morpholino-1,3,5-triazine) derivatives: Potent adenosine 5′-triphosphate competitive phosphatidylinositol-3-kinase/mammalian target of rapamycin inhibitors: Discovery of compound 26 (PKI-587), a highly efficacious dual inhibitor. J. Med. Chem..

[96] Freitag H., Christen F., Lewens F., Grass I., Briest F., Iwaszkiewicz S., Siegmund B., Grabowski P. (2017). Inhibition of mTOR’s catalytic site by PKI-587 is a promising therapeutic option for gastroenteropancreatic neuroendocrine tumor disease. Neuroendocrinology.

[97] Knight S.D., Adams N.D., Burgess J.L., Chaudhari A.M., Darcy M.G., Donatelli C.A., Luengo J.I., Newlander K.A., Parrish C.A., Ridgers L.H. (2010). Discovery of GSK2126458, a highly potent inhibitor of PI3K and the mammalian target of rapamycin. ACS Med. Chem. Lett..

[98] Sutherlin D.P., Bao L., Berry M., Castanedo G., Chuckowree I., Dotson J., Folks A., Friedman L., Goldsmith R., Gunzner J. (2011). Discovery of a potent, selective, and orally available class I phosphatidylinositol 3-kinase (PI3K)/mammalian target of rapamycin (mTOR) kinase inhibitor (GDC-0980) for the treatment of cancer. J. Med. Chem..

[99] Beaufils F., Cmiljanovic N., Cmiljanovic V., Bohnacker T., Melone A., Marone R., Jackson E., Zhang X., Sele A., Borsari C. (2017). 5-(4,6-Dimorpholino-1,3,5-triazin-2-yl)-4-(trifluoromethyl)pyridin-2-amine (PQR309), a potent, brain-penetrant, orally bioavailable, pan-class I PI3K/mTOR inhibitor as clinical candidate in oncology. J. Med. Chem..

[100] Delcuve G.P., Khan D.H., Davie J.R. (2012). Roles of histone deacetylases in epigenetic regulation: Emerging paradigms from studies with inhibitors. Clin. Epigenet..

[101] Rehan M. (2019). Anticancer compound XL765 as PI3K/mTOR dual inhibitor: A structural insight into the inhibitory mechanism using computational approaches. PLoS ONE.

[102] Brown J.R., Hamadani M., Hayslip J., Janssens A., Wagner-Johnston N., Ottmann O., Arnason J., Tilly H., Millenson M., Offner F. (2018). Voxtalisib (XL765) in patients with relapsed or refractory non-Hodgkin lymphoma or chronic lymphocytic leukaemia: An open-label, phase 2 trial. Lancet Haematol..

[103] Chen Y., Yuan X., Zhang W., Tang M., Zheng L., Wang F., Yan W., Yang S., Wei Y., He J. (2019). Discovery of Novel Dual Histone Deacetylase and Mammalian Target of Rapamycin Target Inhibitors as a Promising Strategy for Cancer Therapy. J. Med. Chem..

[104] Yao D., Jiang J., Zhang H., Huang Y., Huang J., Wang J. (2021). Design, synthesis and biological evaluation of dual mTOR/HDAC6 inhibitors in MDA-MB-231 cells. Bioorg. Med. Chem. Lett..

[105] Zhang M., Wei W., Peng C., Ma X., He X., Zhang H., Zhou M. (2021). Discovery of novel pyrazolopyrimidine derivatives as potent mTOR/HDAC bi-functional inhibitors via pharmacophore-merging strategy. Bioorg. Med. Chem. Lett..

[106] Zhai S., Zhang H., Chen R., Wu J., Ai D., Tao S., Cai Y., Zhang J.Q., Wang L. (2021). Design, synthesis and biological evaluation of novel hybrids targeting mTOR and HDACs for potential treatment of hepatocellular carcinoma. Eur. J. Med. Chem..

[107] Bhakuni R., Shaik A., Priya B., Kirubakaran S. (2020). Characterization of SPK 98, a Torin2 analog, as ATR and mTOR dual kinase inhibitor. Bioorg. Med. Chem. Lett..

